# Letheon

**Published:** 1847-10

**Authors:** 


### *The Letheon.*

We take pleasure in informing our readers that Dr. Morton has discontinued selling rights to use the Letheon, and has authorized us to tender the free use of it to every member of the Dental profession in this country, excepting in those places for which he has sold the exclusive right to use it, over which he of course has now no control.

Although not advocates for its use, we think Dr. Morton entitled to the thanks of the Dental profession for his liberality.

We learn that measures are being taken in England to induce the British Government to make him a large grant for his discovery; should this be done, he intends to refund to the purchasers every dollar he has received from the sale of rights.

The best mode of administering the Letheon is to saturate a sponge with pure ether, and allow the patient to inhale the vapor from it; all inhaling instruments are unfit for the purpose, and should be avoided.

STOCKTON & Co., *Dental Intelligencers.*

